# Cone-Beam CT-assisted navigation for endovascular treatment of erection-related artery stenosis in patients with erectile dysfunction

**DOI:** 10.1186/s42155-022-00319-w

**Published:** 2022-08-18

**Authors:** Alexander Rosenov, Nando Mertineit, Iris Baumgartner, Marc Schindewolf

**Affiliations:** 1 Division of Angiology, Swiss Cardiovascular Center, Inselspital, Bern University Hospital, University of Bern, Bern, Switzerland; 2 Department of Radiology, Buergerspital Solothurn, Solothurn, Switzerland

**Keywords:** Erectile dysfunction, ED, Arteriogenic, Vasogenic, Internal pudendal artery, Cone-beam CT, Revascularization

## Abstract

**Background:**

Angioplasty and stenting have emerged as endovascular treatment options for arteriogenic erectile dysfunction over the past few years. Considerable anatomical variation of the erection related pelvic arteries can be challenging during these procedures, leading to time-consuming repetitive super-selective angiograms for navigation.

**Technique:**

We report a novel technique of using C-arm Cone-Beam CT and vessel navigation software to facilitate super-selective catheterization.

**Conclusion:**

Cone-Beam CT-guided navigation for vascular assessment of arteriogenic ED is an optional approach compared to exclusive angiographic assessment. Compared to CT angiography, C-arm Cone-Beam CT offers benefits regarding usage of contrast media and radiation exposure. It has the advantage to combine imaging with endovascular procedures in a single session, reduces time to target navigation in complex pelvic arteries anatomy and may increase therapy safety in endovascular treatment of ED.

## Background

Erectile dysfunction (ED) affects more that 150 million men worldwide and its prevalence increases with the presence of cardiovascular risk factors. These cardiovascular risk factors include age, diabetes, hypertension, dyslipidemia and smoking leading to consecutive endothelial dysfunction, abnormal vasomotion and atherosclerosis (Rogers et al., 2012). Recent imaging studies have shown that 70% to 80% of patients older than 50 years with ED had obstructive lesions of the erection-related arterial system. These findings underline the dominant role of arterial insufficiency in the pathogenesis of ED (Wang et al., 2016; Wang, 2018; Shishehbor & Philip, 2012).

Safety and feasibility of endovascular therapy of erection-related arteries in patients with arteriogenic ED was repeatedly demonstrated over the past few years (Rogers et al., 2012; Wang et al., 2014; Diehm et al., 2019). Balloon angioplasty and/or stenting is usually reserved for patients with suboptimal response to phosphodiesterase 5 inhibitors, which are considered first line therapy (Rogers et al., 2012; Wang et al., 2016; Wang et al., 2014; Diehm et al., 2018). A great variability of the pelvic vascular anatomic features has been reported as main challenge of these endovascular procedures (Rogers et al., 2012; Wang et al., 2014; Kawanishi et al., 2008). As an alternative to solely angiographic evaluation, contemporary reports have documented cross-sectional imaging, mainly CT angiography, for procedure planning (Wang et al., 2016; Diehm et al., 2019; Diehm et al., 2018). We report the application of C-arm Cone-Beam CT (CBCT) and vessel navigation software for this indication.

## Technique

**Fig. 1:**
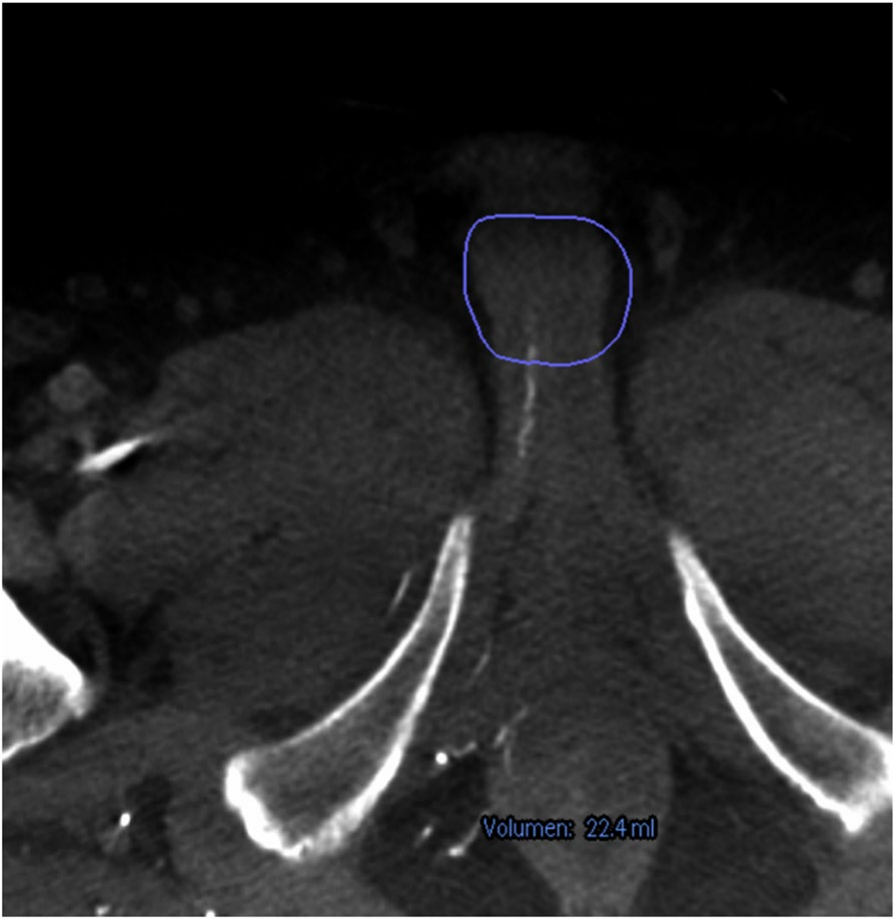
After performing a cone-beam computed tomography (CBCT) the EmboGuide® software (Philips) is used to calculate a road map. As a first step the root of the penis is marked as navigation target (blue circle). Shown are the images of a 63-year-old patient with arteriogenic erectile dysfunction

**Fig. 2:**
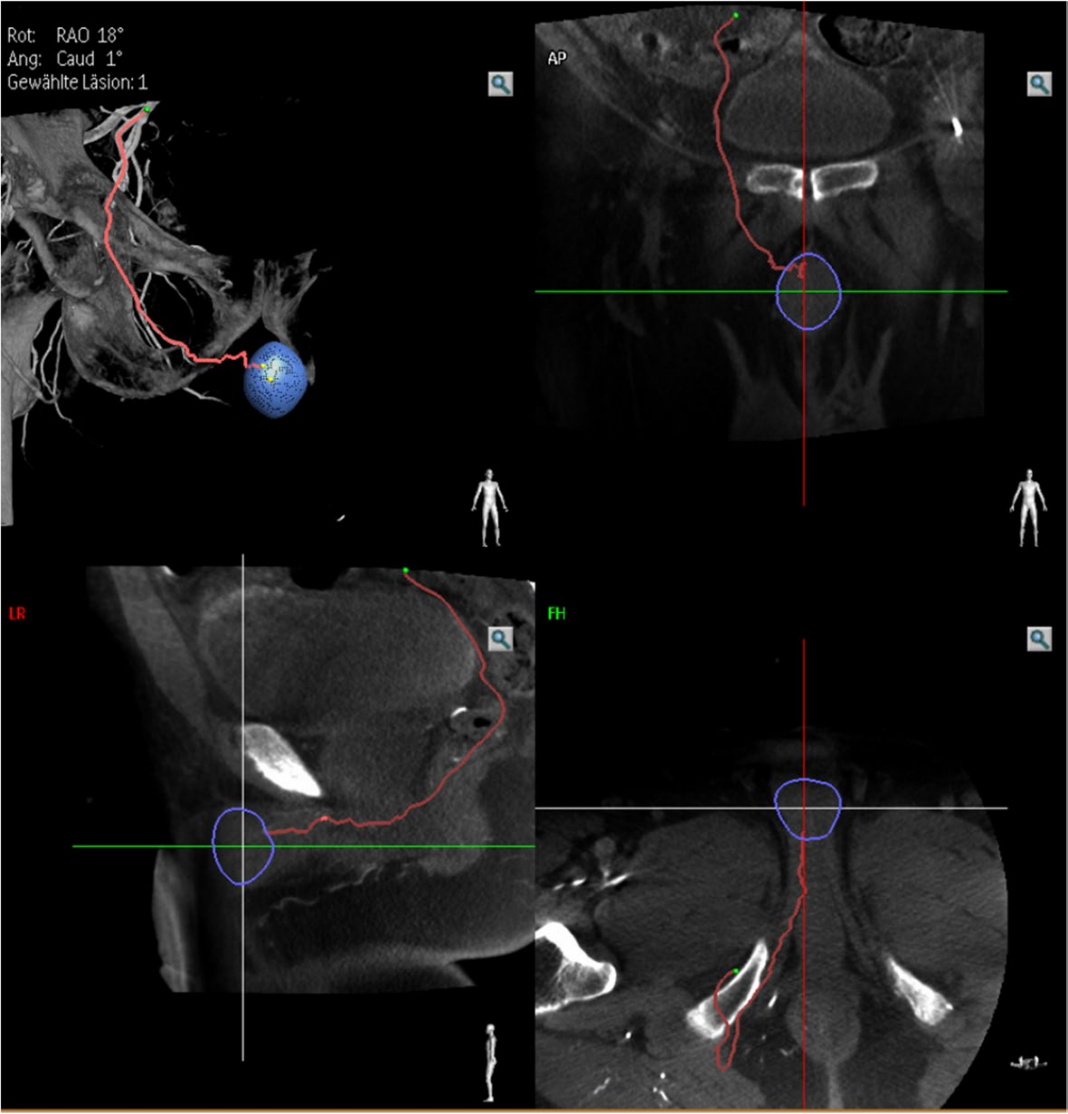
A three-dimensional road map from the tip of the diagnostic catheter to the navigation target (blue circle) is created by the software

**Fig. 3:**
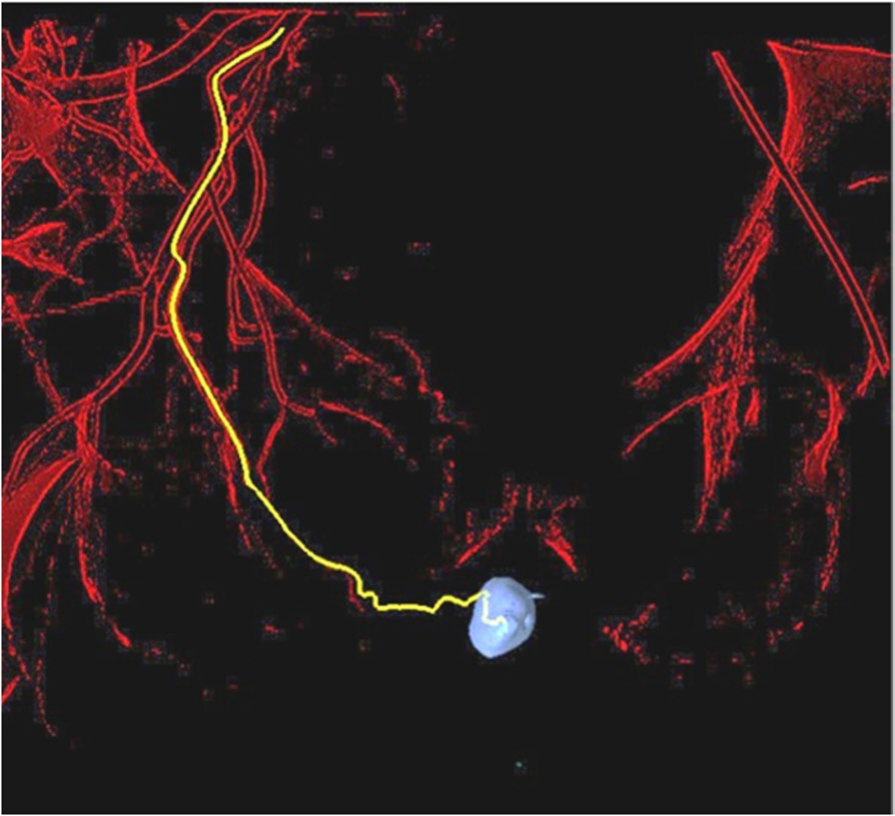
The calculated roadmap can now be used as three-dimensional overlay

**Fig. 4:**
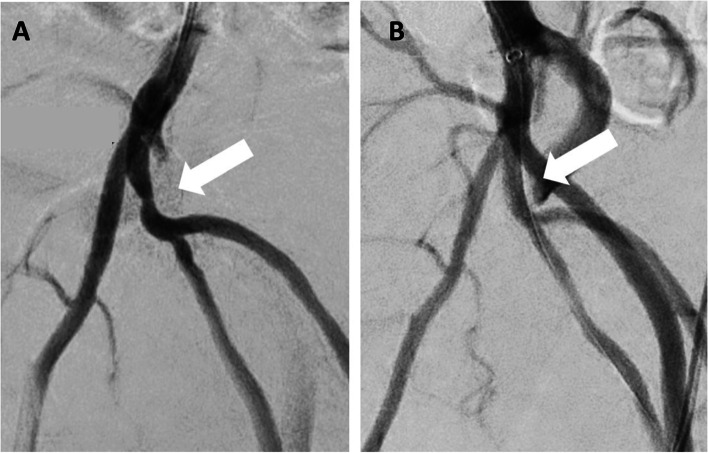
Stenosis of the right internal pudendal artery before (**A**) and after (**B**) stenting in the same patient

In our practice, patients with erectile dysfunction and documented impaired arterial perfusion in penile duplex sonography, who are insufficiently responding to medical treatment, are candidates for further evaluation of vascular assessment and therapy. If the decision for endovascular revascularization is made, we usually schedule a diagnostic angiography of the erection related iliac arteries. In case an obvious interventional target of large arteries is not visible in a selective angiogram of the hypogastric artery, a cone beam CT (Philips, Best, Netherlands) can be performed using an 8 seconds arterial scanning protocol. In this case we inject 40 ml of diluted (ratio 1:1 with isotonic saline) contrast agent (Iopamiro®, Bracco Suisse SA, Cadempino, TI, Switzerland) with an injection flow of 4 ml/sec and 2.5 seconds X-ray delay via a diagnostic catheter placed in the hypogastric artery. Data acquisition and processing is performed using EmboGuide® navigation software (Philips). The root of the penis and the tip of the diagnostic catheter are set as markers for software-automated vessel track calculation and construction of a 3-dimensional modelling of the vascular tree (Figs. 1, 2 and 3). Quick selective target catheterization down the erection-related artery path using the 3-dimensional vascular overlay can now be conducted using a 2.7 F microcatheter (Progreat®, Terumo, Tokyo, Japan). Often, repetitive small dosages of intra-arterial nitroglycerin boli are applied allowing the assessment of the smallest distal arteries (Perlinganit®, CPS Cito Pharma, Uster, ZH, Switzerland) and for identifying the culprit lesion. Depending on the lesion diameter and length drug-coated balloon angioplasty or drug-eluted stenting is performed (Fig. 4). We prescribe a dual antiplatelet treatment with aspirin 100 mg and clopidogrel 75 mg daily for 12 months. A follow up consultation is planned 4 weeks after the procedure.

## Discussion

Selective angiographic assessment of the erection-related arteries solely guided by conventional DSA can be challenging and time consuming due to underlying variable pelvic artery anatomy (Rogers et al., 2012; Wang et al., 2014). While proximal stenosis of the internal iliac artery or the internal pudendal artery are usually detected by conventional angiography, the absence of proximal stenoses requires superselective assessment of the more peripheral arteries, especially when pre-interventional penile duplex ultrasound indicates arterial insufficiency. Anatomic variation of pelvic arteries is not only an impediment for superselective catheterization but also for identifying erection-related arteries. Accordingly, unintentional non-target vessel stenting has been reported in 17% of patients in the ZEN study (Rogers et al., 2012).

C-arm Cone-Beam computed tomography is integrated in many modern angiographic systems and allows cross-sectional imaging and the possibility to use dedicated planning and navigation software (Floridi et al., 2014). Established clinical applications for CBCT and vessel navigation software are embolization therapies for tumors, arterial bleedings (Grosse et al., 2018; Tacher et al., 2015), and revascularization of pulmonary arteries in patients with chronic thromboembolic pulmonary hypertension (Sugiyama et al., 2014). In our case, we applied current imaging and navigation techniques to evaluate the vascular anatomy and create a 3D roadmap to reduce time to target lesion and to increase interventional safety.

Compared to non-invasive three-dimensional volumetric imaging workup, as CT Angiography (CTA) and MR-Angiography (MRA), CBCT allows of visualizing, planning and treating complex arteriogenic lesions of pelvic arteries in a single session. Compared to CTA, CBCT displays vascular anatomy more accurate and with a higher resolution in smaller and more peripheral arteries due to the direct intra-arterial contrast administration (Tacher et al., 2015). The amount of iodinated contrast medium used in our case was less than 25% of a traditional abdominal CTA in our hospital (CBCT 20 ml Iopamiro® vs. CTA 100 - 120 ml Ultravist 370®, Bayer (Schweiz) AG, ZH, Switzerland). The radiation exposure of a conventional abdominal CTA exceeds an abdominal CBCT by 3–4 times (Tacher et al., 2015). However, as the image section of CBCT usually does not cover the aorta to the root of the penis due to detector size, two selective CBCTs with the diagnostic catheter placed in the internal iliac artery have to be performed, one for each side. This reduces the radiation and contrast saving benefits of CBCT over CTA. Contrariwise, there have been promising developments in MRA, which causes no radiation exposure and does not necessarily require intravenous contrast agent. However, visualization of small, peripheral arteries using MRI techniques is still challenging, especially in tortuous vessels and if motion artifacts come into play, as in the pelvis (Mathew & Kramer, 2018). As conventional MRA using gadolinium based contrast agents still is a frequently used technique, potential systemic effects of gadolinium-based contrast agents also play a relevant role in decision making (Mathur et al., 2020). On top of this, limited availability and high costs are also worthwhile considering. The main disadvantage of on table cone beam CT application compared to CTA or MR-Angiography is that a diagnostic catheter needs to be placed selectively. Nevertheless, appropriate pre-interventional non-invasive diagnostic imaging workup, i.e. duplex ultrasound is required for correct planning of invasive procedures such as CBCT. If duplex ultrasound and the clinical symptoms support arteriogenic pathogenesis of ED, an invasive assessment with the option to treat stenotic lesion in the same session is the recommended diagnostic and therapeutic strategy.

## Conclusion

In cases with suggestive arteriogenic ED, CBCT-guided navigation for vascular assessment of arteriogenic ED is an optional approach compared to exclusive angiographic assessment. Compared to CTA, CBCT offers benefits regarding usage of contrast media and radiation exposure. It has the advantage to combine imaging with endovascular procedures in a single session, reduces time to target navigation in complex pelvic arteries anatomy and may increase therapy safety in endovascular treatment of ED.

## Data Availability

Data sharing is not applicable as no datasets were generated or analyzed for this publication.

## Abbreviations

ED: Erectile dysfunction
CT: Computed tomography
CBCT: C-arm cone beam computed tomography
PSV: Peak systolic velocity
EDV: End diastolic velocity
IIEF: International index of erectile dysfunction
DSA: Digital subtraction angiography
CTA: Computed tomography angiography
MRA: Magnetic resonance angiography
MRI: Magnetic resonance imaging
